# Extracellular Matrix Density Regulates the Rate of Neovessel Growth and Branching in Sprouting Angiogenesis

**DOI:** 10.1371/journal.pone.0085178

**Published:** 2014-01-22

**Authors:** Lowell T. Edgar, Clayton J. Underwood, James E. Guilkey, James B. Hoying, Jeffrey A. Weiss

**Affiliations:** 1 Department of Bioengineering, University of Utah, Salt Lake City, Utah, United States of America; 2 Scientific Computing and Imaging Institute, University of Utah, Salt Lake City, Utah, United States of America; 3 Medical Products Division, W.L. Gore and Associates, Inc., Flagstaff, Arizona, United States of America; 4 Department of Mechanical Engineering, University of Utah, Salt Lake City, Utah, United States of America; 5 Division of Cardiovascular Therapeutics, Cardiovascular Innovation Institute, University of Louisville, Kentucky, United States of America; UT-Southwestern Med Ctr, United States of America

## Abstract

Angiogenesis is regulated by the local microenvironment, including the mechanical interactions between neovessel sprouts and the extracellular matrix (ECM). However, the mechanisms controlling the relationship of mechanical and biophysical properties of the ECM to neovessel growth during sprouting angiogenesis are just beginning to be understood. In this research, we characterized the relationship between matrix density and microvascular topology in an *in vitro* 3D organ culture model of sprouting angiogenesis. We used these results to design and calibrate a computational growth model to demonstrate how changes in individual neovessel behavior produce the changes in vascular topology that were observed experimentally. Vascularized gels with higher collagen densities produced neovasculatures with shorter vessel lengths, less branch points, and reduced network interconnectivity. The computational model was able to predict these experimental results by scaling the rates of neovessel growth and branching according to local matrix density. As a final demonstration of utility of the modeling framework, we used our growth model to predict several scenarios of practical interest that could not be investigated experimentally using the organ culture model. Increasing the density of the ECM significantly reduced angiogenesis and network formation within a 3D organ culture model of angiogenesis. Increasing the density of the matrix increases the stiffness of the ECM, changing how neovessels are able to deform and remodel their surroundings. The computational framework outlined in this study was capable of predicting this observed experimental behavior by adjusting neovessel growth rate and branching probability according to local ECM density, demonstrating that altering the stiffness of the ECM via increasing matrix density affects neovessel behavior, thereby regulated vascular topology during angiogenesis.

## Introduction

Angiogenesis is the generation of new vascular elements from existing vasculature. During angiogenesis, sprouting endothelial cells degrade the basement membrane with matrix metalloproteinases (MMPs) [1], [2] and apply traction to and migrate along components of the extracellular matrix (ECM) [3], resulting in neovessel elongation. Previous studies have demonstrated that the mechanical interaction between neovessel sprouts and the ECM regulate the topology of vascular networks formed during angiogenesis [4]–[7]. Cellular traction forces applied to the ECM create a deformation that cells can detect [8]–[10]. The nature of this deformation is determined by the material properties of the matrix, such as fibril orientation and density, as well as geometry and boundary conditions. In previous studies, we explored the relationship between angiogenesis and the mechanical properties of the ECM using a three-dimensional (3D) organ culture model of microvessel fragments within a type-I collagen gel [11]–[14]. In this model of sprouting angiogenesis, neovessels sprouting from whole microvessels cultured in a 3D gel, that was free to contract in all directions, grow into a randomly oriented network [12]–[14]. When contraction was prevented along the long-axis of rectangular gels, neovessels and collagen fibrils were aligned parallel to the constrained axis [13], [14].

In addition to the effects of mechanical boundary conditions, ECM matrix density has been shown to influence formation of capillary structures in various *in vitro* models of angiogenesis. Several studies utilizing endothelial cell-based culture models of angiogenesis have shown that increasing the density of the matrix reduces capillary outgrowth and network formation [15]–[20]. However, all of these studies involved models of outgrowth from isolated endothelial cells. In contrast, our microvessel organ culture model of angiogenesis is based on intact microvessels and includes multicellular interactions between vascular cells (endothelial and mural cells) and the nascent matrices (basement membrane and stroma). Additionally, previous studies only reported the effect of matrix density increase using quantitative data measured over the capillary network and as a result were only able to report global changes to the topology of the network. As a result, these studies were not able to establish a strong connection between their global experimental observations and local cellular behavior.

Experimental cell and organ culture models of angiogenesis typically provide morphometric data that are obtained by averaging information about the entire culture, at a single time point. This makes it difficult to establish a relationship between local cellular behavior and global characteristics of the biological system. Computational models can supplement and enhance the interpretation of experimental results, and are commonly utilized within the field of angiogenesis research [21]. Recently, we developed a computational model of angiogenesis that uses the orientation of ECM fibrils to determine the direction of microvessel growth and produced validated predictions of global morphometric data when compared to *in vitro* vascular networks [22]. The goal of this research was to extend and apply this model to characterize the response of angiogenic microvessels to changes in matrix density, using a combined experimental and computational approach. First, we performed experiments using our 3D angiogenesis model with different type-I collagen densities to characterize the relationship between vascular topology and matrix density. Second, we used our experimental data to design and calibrate new features within our growth model to describe how neovessels respond to a change in matrix density. Finally, we demonstrated how this response leads to the changes in vascular topology that we found.

## Materials and Methods

### In vitro model of angiogenesis

**Figure 1:**
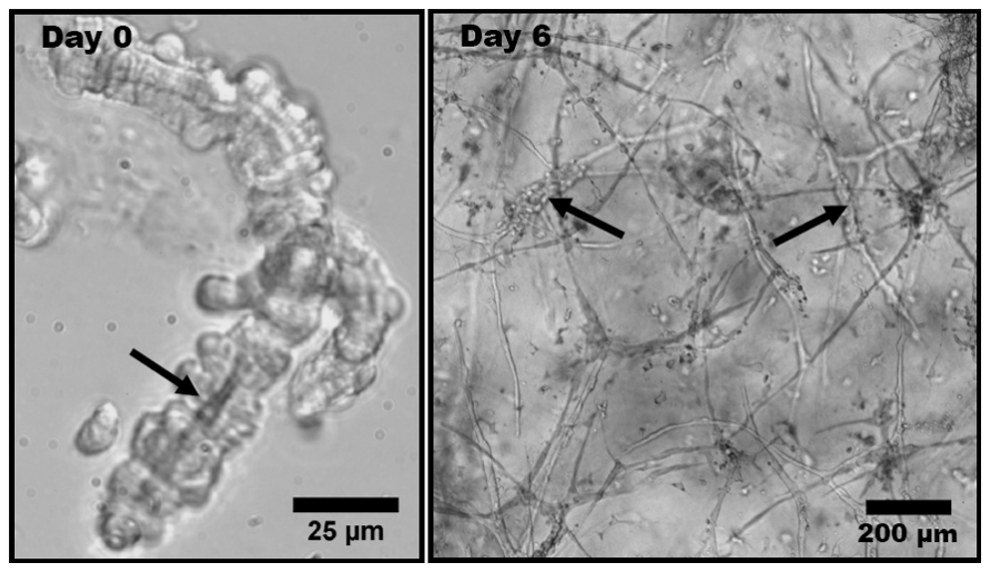
Phase contrast light micrographs of rat microvessel fragments. (Left) Isolated microvessel fragment at Day 0 with a visible lumen, indicated by the arrow. (Right) Angiogenic microvessel fragments within a type I collagen gel at Day 6 of growth. The thicker initial fragments are indicated by the arrows. The thinner protrusions extending from the initial fragments are neovessels formed through angiogenesis.

3D vascularized constructs served as a model of *in vitro* angiogenesis and were prepared using methods described previously [11]. Constructs consisted of microvessel fragments isolated from rat epidydymal fat pads, resuspended and polymerized in 3D collagen gels (Fig. 1). The microvessel fragments retain the associated stromal cells and basement membrane after isolation and seeding, providing an “organ culture” model of angiogenesis. Previous studies have extensively characterized angiogenesis in these vascularized constructs [11]–[13], [23]–[28]. Sprouting of neovessel tips occurs spontaneously and predictably, with the first evidence of sprout tips usually observed by the second day of culture.

All reagents were obtained from Invitrogen (Carlsbad, CA) unless otherwise indicated. Epididymal fat pads were harvested from male retired breeder Sprague-Dawley rats. This protocol was approved by the University of Utah Institutional Animal Care and Use Committee (Protocol Number: 11-02018). Fat pads were minced and subjected to partial digestion with 2.0 mg/ml Clostridium collagenase (Worthington Biochemicals, Lakewood, NJ). After four minutes, digestion was halted by the addition of Leibowitz (L-15) media and the solution was centrifuged. The pellet was washed, resuspended in media, and filtered through 350 µm and 30 µm sterile nylon filters sequentially. The filtration step removes undigested debris, single cells, and small fragments resulting in a population of microvessel fragments within a controllable range of sizes. At 35,000 per ml, vessel fragments were suspended within liquid type-I collagen solution (BD Biosciences, Bedford, MA) prepared at concentrations of 2.0, 3.0, or 4.0 mg/ml. For each of the three collagen solutions, 0.5 ml was transferred to a circular culture well (Nunc – Thermo Fisher Scientific, Rochester, NY, diameter = 15 mm) and allowed to polymerize into a three-dimensional gel (n = 4 gels per collagen concentration investigated, N = 12 gels total). These gels were free-floating within the culture chambers with initial dimensions of 15 mm diameter and 2.8 mm thick.

Serum-free growth media [29] was supplemented with 10.0 ng/ml rhVEGF (PeproTech, Rocky Hill, NJ) and provided to the cultures at Day 0. Media was changed every two days and replaced with 0.8 ml of fresh media. After Day 6 of culture, the gels were fixed with 1.0 ml of 4% paraformaldehyde for 24 hours. Gels were then placed in a solution of 1× phosphate-buffered saline (DCF-PBS, pH 7.4) and 2.0 µg/ml Isolectin IB4-Alexa 488 to fluorescently label endothelial cells in preparation for confocal microscopy.

### Confocal microscopy and skeletonization of vascular networks

**Figure 2:**
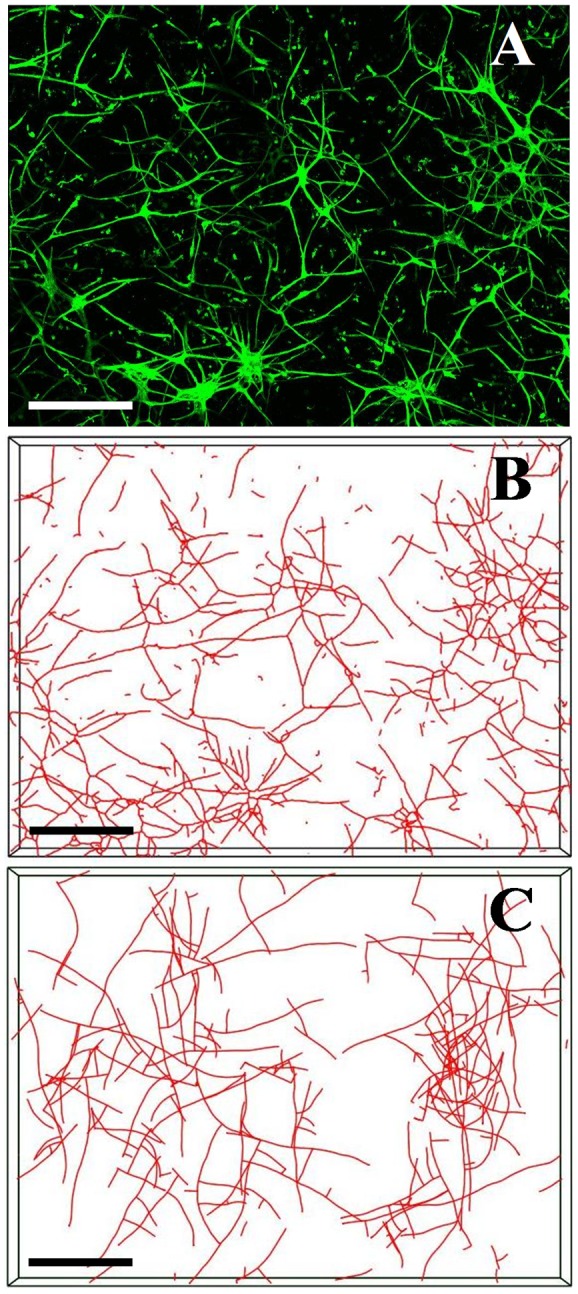
Visualization of *in vitro* experiments and simulation results. The computational model was designed to simulate angiogenic outgrowth and neovascularization within 3D organ culture of microvessel fragments with a type-I collagen gel. All images in this figure depict the 3.8×2.5×0.2 mm region that was imaged during the experiments.(A) Z-projection mosaic of 3D confocal image data showing microvessels cultured in a 3.0 mg/ml collagen gel after Day 6 of culture. Endothelial cells within the culture were labeled with Isolectin IB4-Alexa 488 and imaged using a confocal microscope with a 10× objective. (B) Skeletonized vessel data obtained from the confocal image data of a vascularized collagen gel in Panel A. (C) Results of a simulation using the computational model. Microvessels were represented as a collection of line segments, and growth was simulated by the addition of new segments to the free ends of existing segments. (Scale bar = 350 µm).

Three-dimensional image data were collected from each vascularized construct by confocal microscopy using methods described previously [12], [13]. Six adjacent stacks at the geometric center of each gel were captured with an Olympus FV1000 microscope using a 10× objective and 488 nm laser. Each stack was captured at a resolution of 512×512 pixels and was acquired to a depth of 200 µm at 2.5 µm intervals. A 2×3 mosaic was created from the six stacks using a custom software application, resulting in an image dataset that represented a 3.8×2.5×0.2 mm region of the vascularized gel (Fig. 2 *A*). Processing and skeletonization of 3D confocal data was performed using the Amira™ software (Mercury Computer Systems, Carlsbad, CA). Mosaic stacks were subjected to a deconvolution routine to reduce out-of-plane blur through the depth of the dataset (numerical aperture = 0.4, wavelength = 520 nm, refractive index of collagen = 1.35 [30]). A threshold value was calculated by fitting a Gaussian distribution to the image histogram, and this value was used in the automated skeletonization routine within Amira to decompose images of vessels down to a collection of line segments (Fig. 2 *B*). A custom software application was used to analyze the skeletonized vessel data and to collect the desired morphometric data [31]. The image data of the vascularized constructs were analyzed to determine the *total vascular length*, the degree of *network interconnectivity*, the number of *branching points*, and the number of *end points*.

The collective length of all microvessels within the imaged domain was defined as the *total vascular length*. This morphometric measurement can also be interpreted as the contour length of the network, and it is a useful metric of overall angiogenic outgrowth during the culture period [12], [13]. Neovessels sprouting from parent fragments were observed to form continuous vascular networks, with inter-vessel connections presumably formed through anastomosis. The *network interconnectivity*, or the percentage of microvessels that fused into a single continuous vascular network, was calculated by measuring the length of the longest continuous microvessel tree and normalizing by the total vascular length. A *branching point* was defined as a node connected to 3 different line segments and indicates either a new vessel sprout (branching) or two separate vessels fusing into one (anastomosis). *End points* were terminal ends of vessels, defined by a node that is associated with only one segment. The number of end points quantifies the amount of growth tips and characterizes the degree of branching and anastomosis occurring within the domain.

### Computational growth model

A validated computational model of microvessel growth and 3D vessel morphology during spontaneous angiogenesis was utilized to simulate the *in vitro* experiments (Fig. 2 *C*) [22]. This growth model uses information about the ECM to predict neovessel length, direction, and branching. A simulation domain corresponding to the imaged region of each vascularized gel (3.8×2.5×0.2 mm) was fit with a regular hexahedral grid at a resolution of 6×6×2 elements. Properties of the ECM that influenced angiogenic vessel growth, such as fibril orientation and matrix density, were stored at the nodes of the grid. The model was initialized with a random collagen fibril orientation value between 0 and π radians at each node in the grid. Initial microvessel fragments were represented as line segments and were seeded at random positions and orientations throughout the domain.

At each time step, microvessel growth was simulated by creating new line segments at the positions of all active growth tips based on methods presented previously [22]. A unit vector, ***ψ*** *_new_*, was used to describe the direction of each new line segment. Its orientation was determined by a weighted average of the direction of the parent microvessel, represented by the unit vector ***ψ*** *_parent_*, and a directional component determined by the local collagen fibril orientation, represented by the unit vector ***θ***:
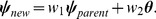
(1)The values of *w_1_* = 0.91 and *w_2_ = *0.09 were determined based on preliminary simulations to create microvascular networks with similar morphology to those seen *in vitro*. The component of neovessel direction determined by collagen fibril guidance, ***θ***, was interpolated using trilinear finite element shape functions through the following equation:
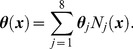
(2)In Eq. (2), ***θ*** *_j_* is the collagen fibril orientation value stored at node *j* of the grid element in which the growth tip resides. *N* *_j_* is the value of the shape function for node *j* at the position of the active growth tip, ***x***. The shape functions, *N*(***x***), provide an interpolation scheme in which the value of the field variable at a point in the grid element is a weighted average of contributions from each node.

Branching, the sprouting of a new neovessel from an existing vessel, was modeled as a random process as per our previous publication [22]. During a time step, each segment generated a random number between 0 and 1. If the random number exceeded the branching probability, *b_0_*, then that segment would form a branch. Similarly, the growth rule for anastomosis, the fusing of two vessels into one, followed our previous approach [22]. If a growth tip was within 25 µm of another vessel, anastomosis was simulated by creating a new line segment connecting the two points. Subsequently, all growth tips involved with the anastomosis were inactivated and stopped growing.

To introduce a sensitivity of vessel growth to matrix density, a scaling factor was calculated from experimental data by taking the average total vascular length for each culture condition (2.0, 3.0, and 4.0 mg/ml) and normalizing by the average total vascular length measured in the 3.0 mg/ml constructs. Values of the scaling factor *v* were then fit to a three-parameter exponential function that described how the factor changed with collagen density:
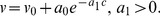
(3)Here, *v* was the scaling factor, *c* was the collagen concentration (mg/ml), and *v_0_*, *a_0_*, and *a_1_* were parameters determined by the curve fit. An additional data point of *c* = 10.0 mg/ml, *v* = 0.0 was added to ensure that the scaling function eventually reached zero. The collagen concentration of the matrix was an input parameter to the computational model and the corresponding scaling factor was calculated using Eq. (3). The scaling factor was used to proportionally scale two growth mechanisms. As the density of the matrix increased, the scaling factor reduced the length of new line segments and probability of each segment forming a branch.

Simulations of the *in vitro* experiments were performed in order validate the computational framework (4 simulations per culture condition, 12 simulations total). The number of initial fragments in the simulations (*N_frag_* = 70) was determined from the volume of the domain and the seeding density (35,000 fragments per ml). Growth was simulated for six days. The branching probability was set at 0.1, determined by minimizing the RMS error between the number of branch points in simulations and experiments at 3.0 mg/ml using methods previously described [22]. The collagen concentration used to prepare the gel experiment being simulated (2.0, 3.0, or 4.0 mg/ml) was set as an input parameter read in by the model during initialization. For each culture condition, morphometric data from the computational simulations (total vessel length, network interconnectivity, number of branch points, and number of end points) were compared to values measured from the corresponding experimental cultures.

### Statistical Analysis

One-way ANOVAs were used to test for the effect of matrix density on all morphometric parameters for both experimental and simulation data (*α* = 0.05). A two-tailed Student's T-test with unequal variance (Welch's T-test) was then performed between experimental and computational morphometric data for each matrix density to detect any statistical difference (*α* = 0.05). If a statistical difference could not be detected, a TOST-test (Two One-Sided T-test [32]) was performed to test for statistical equivalence (*α* = 0.05, *Θ* = 0.3).

### Predictive Simulations

Single cell experiments involving micropatterned ECM have been useful in studying the role of haptotaxis (guidance by ligand availability) and durotaxis (guidance by substrate stiffness/deformability) in cell migration. Similar experiments involving tailored ECM density fields would prove useful in studying the mechanical regulation of angiogenesis. However, our organ culture method involves encapsulating microvessel fragments within a collagen gel, preventing us from growing these vessels in 3D pre-fabricated matrices. However, our computational framework can be used to predict the results of the type of experiments that we cannot perform using our current culture method. As a final demonstration of utility of the modeling framework, we used our growth model to predict several scenarios of practical interest that could not be investigated experimentally.

In the first predictive simulation, a density gradient was created within the simulation domain (7.6×5.1×0.4 mm). The gradient ran along the longest axis of the domain (*x*-axis) from 1.0 mg/ml at the left edge to 10.0 mg/ml at the right edge. Matrix density was uniform along the other two directions. This simulation demonstrates how angiogenic growth changes as neovessel grow up or down a stiffness gradient. In the second predictive simulation, a cylindrical plug (radius = 1.5 mm) composed of acellular 10.0 mg/ml collagen was placed in the center of the domain. The remaining domain was initialized at 3.0 mg/ml and seeded with vessels. This simulation demonstrates how neovessels respond to a stiff interface as they encounter the acellular plug. The final predictive simulation demonstrates how vasculature can be aligned along a chosen direction by growing neovessels within two 1000 µm channels of 3.0 mg/ml collagen running along the *x*-axis of the domain. The density outside the channels was set to 8.0 mg/ml. Vessels were seeded within the channels only. In all three of the predictive simulations, vessels were seeded at a density of 10,000 fragments per ml and growth was simulated until Day 6.

## Results

### Experimental Results

**Figure 3:**
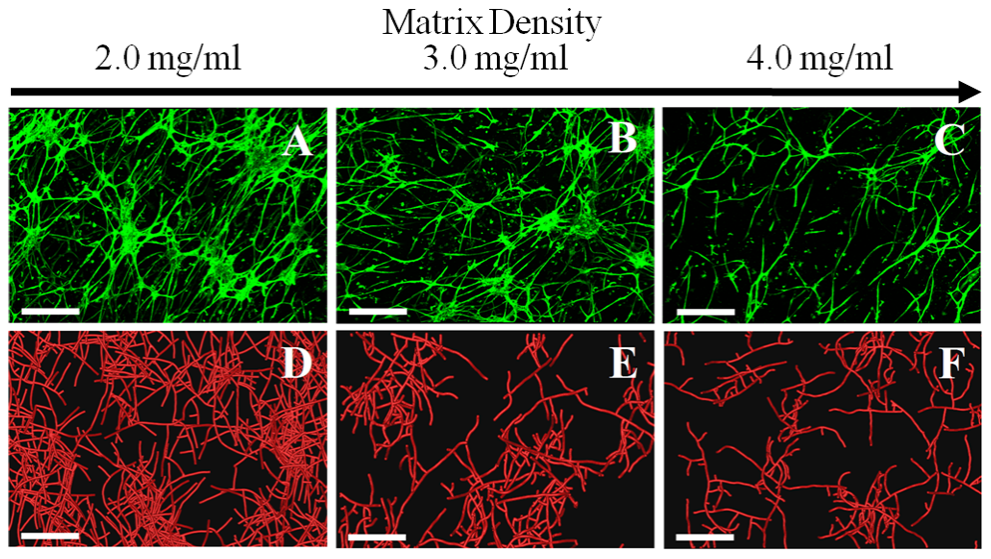
Microvasculatures observed at different levels of collagen density. Increasing the density of the ECM reduced neovascularization in both the experiments and computational simulations. Top Row: Z-projection mosaic of 3D confocal image data showing vascularized collagen gels taken at Day 6 of growth. Bottom Row: Results of the comparable computational simulations, presented as 3D volume-renderings of the line segment data. The three levels of collagen density assessed in this study were: 2.0 mg/ml (A, D), 3.0 mg/ml (B, E), and 4.0 mg/ml (C, F). (Scale bar = 350 µm).

Angiogenic sprouting and neovessel outgrowth was consistent across all microvessel cultures. A well-established microvasculature was observed at Day 6 for all matrix density conditions. The circular gels experienced significant contraction during the six-day culture period, with construct diameters reduced by an average of 40.0±5.0%, 20.0±5.0%, and 7.0±1.0% for 2.0, 3.0, and 4.0 mg/ml gels, respectively (average ± std dev). Qualitatively, the highest levels of angiogenic growth occurred in the 2.0 mg/ml constructs, while growth within the 4.0 mg/ml constructs appeared considerably reduced (Fig. 3 *A–C*).

**Figure 4:**
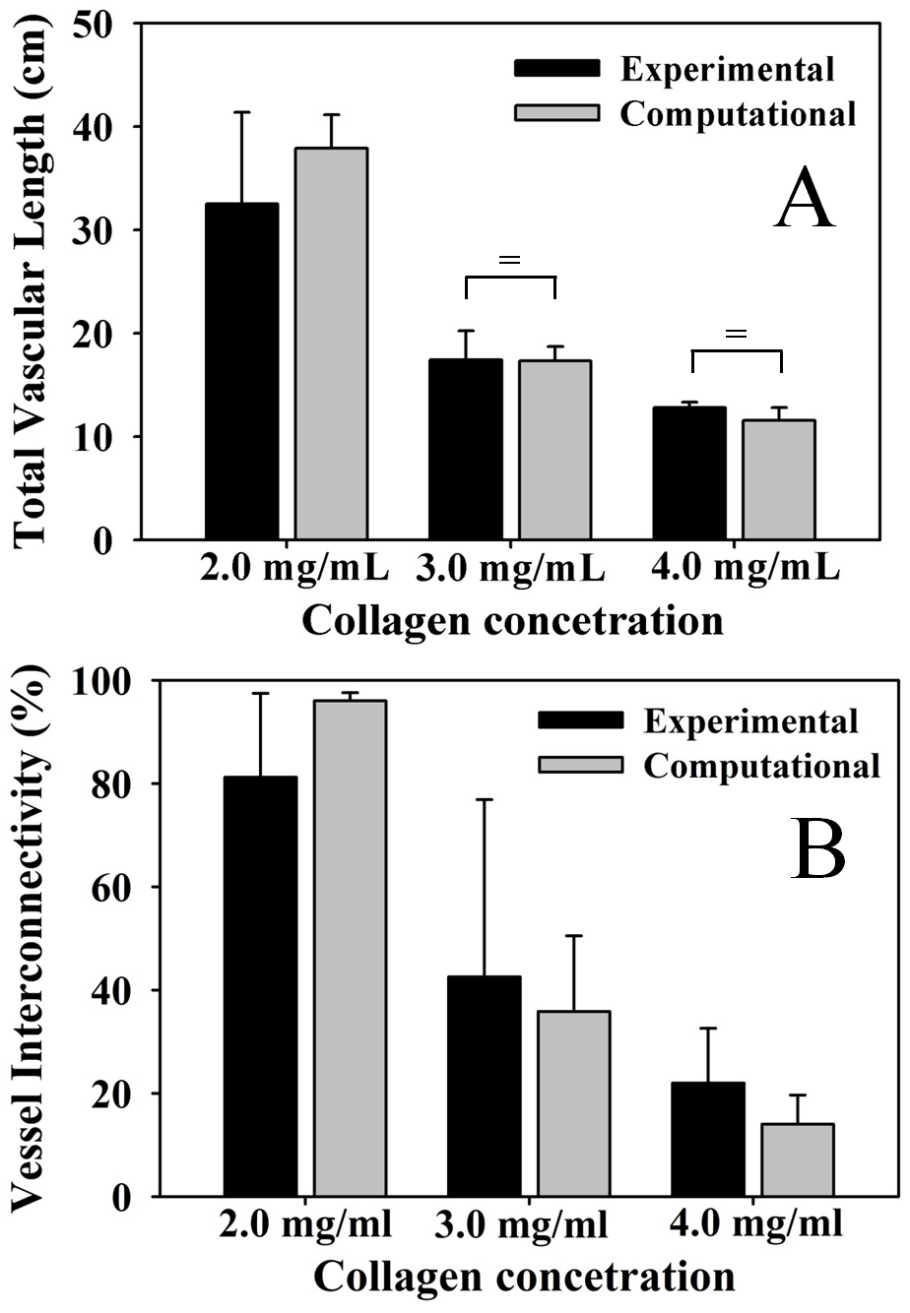
Total vascular length and network interconnectivity. (A) The total vascular length decreased as matrix density increased. Measurements from the experimental cultures are presented in black and predictions from the computational model are presented in gray. (B) Vessel interconnectivity, a measure of the percentage of microvessels within the domain that are connected into the largest continuous vascular network, decreased as a function of matrix density, indicating a reduction in network formation. There was a significant effect of matrix density on total vascular length and network connectivity for both experimental and computational results (One-way ANOVA, p<0.05 in all 4 cases). No statistical difference was detected between any experimental and computational morphometric at each matrix density level by T-test. Statistical equivalence as detected by a TOST-test is indicated by the bracket and equal sign.

Measurements of total vascular length within the vascularized constructs indicated a reduction in overall neovessel outgrowth as the density of the matrix was increased (Fig. 4 *A black*) (ANOVA, P<0.05). Although we did not measure the initial total vascular length in these experiments, data from a previously published study [22] allows us to approximate the initial total vascular length for these cultures at 2.7 cm. This allows us to determine that over the 6 days of culture, total vascular length experienced roughly an 11-fold, a 6-fold, and a 4-fold increase in the 2.0, 3.0, and 4.0 mg/ml gel experiments respectively. There was a significant decrease in network connectivity as the matrix density increased (Fig. 4 *B black*) (ANOVA, P<0.05), indicating that increased density of the matrix results in a more discontinuous vascular network.

**Figure 5:**
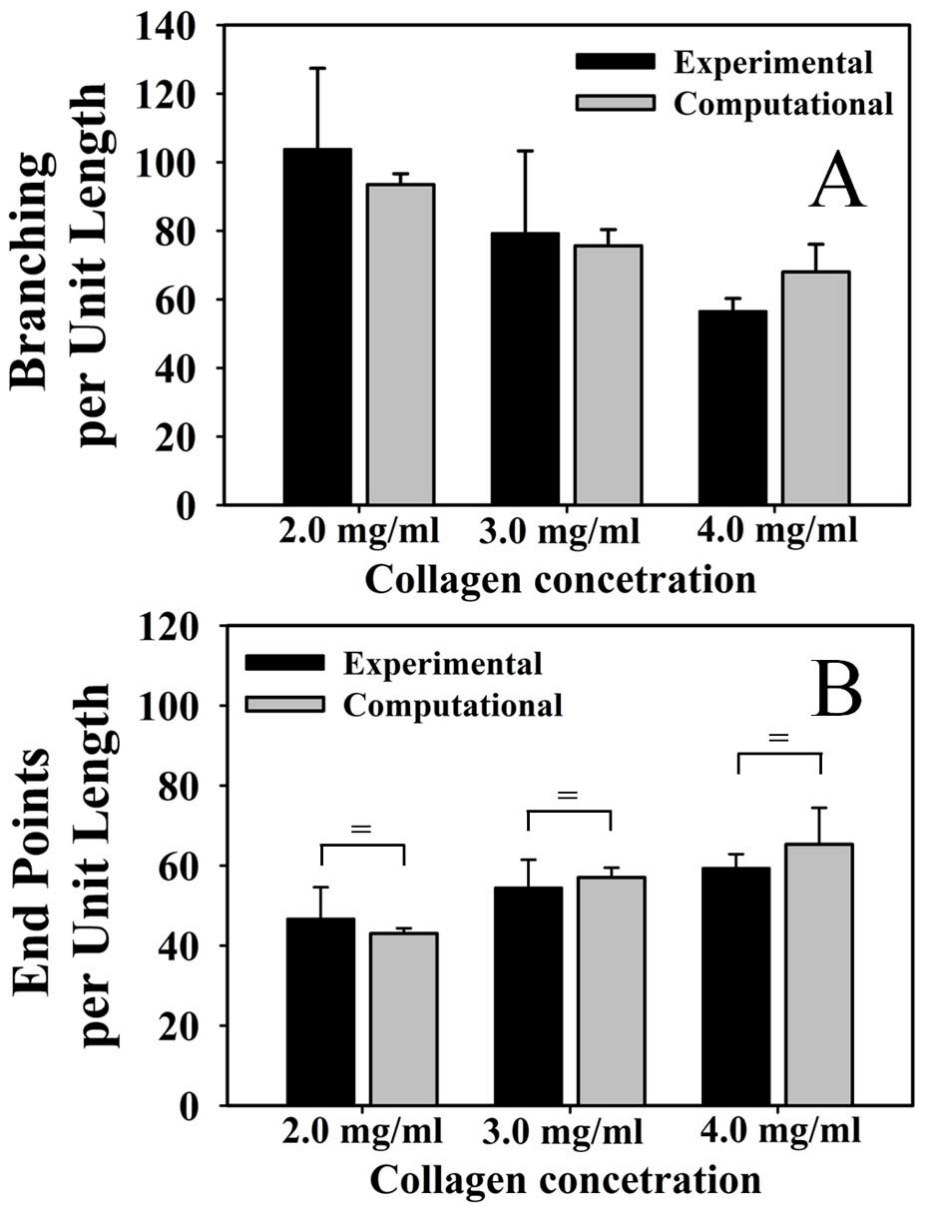
Branch points and free ends per unit length. A branching point was defined as any node that connected to three or more vessel segments. Branching points were created by either a new vessel sprout (branching) or two separate vessels fusing into one (anastomosis). Measurements from the experimental cultures are presented in black and predictions from the computational model are presented in gray. (A) The number of branch points was normalized by the total vascular length in order to isolate the tendency of microvessels to form a branch point per unit length of growth. Branching per unit length was observed to decrease as matrix density was increased. An end point was defined as a node that was associated with only one vessel segment and represents the terminal end of a vessel. Measurements from the experimental cultures are presented in black and predictions from the computational model are presented in gray. (B) Normalizing the number of end points by the total vascular length revealed that the number of free ends per unit length increased along with matrix density. There was a significant effect of matrix density on branch points and free ends per unit length for both experimental and computational results (One-way ANOVA, p<0.05 in all 4 cases). No statistical difference was detected between any experimental and computational morphometric at each matrix density level by T-test. Statistical equivalence as detected by a TOST-test is indicated by the bracket and equal sign.

Other angiogenic neovessel behaviors, such as branching and anastomosis, were characterized by measuring the number of branch points and end points within the vascularized constructs. At Day 6 of culture, there was a significant decrease in the number of branch points and end points as the collagen density increased (data not shown). Since increased contraction of low-density gels could lead to increased branch points and end points within an imaged volume, branch point and end point data were normalized by total vascular length to express these measurements per unit length of growth. As the density of the matrix increased, microvessels formed significantly less branch points per unit length of growth (Fig. 5*A black* ) (ANOVA, P<0.05). The amount of free ends per unit length increased significantly as matrix density increased (Fig. 5 *B black*) (ANOVA, P<0.05).

### Computational Results

**Figure 6:**
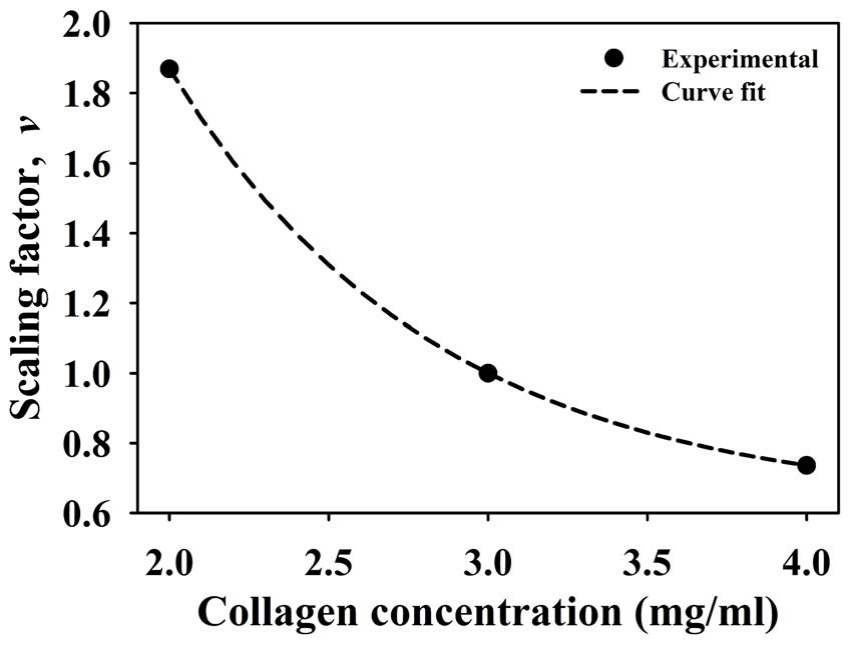
Matrix density scaling factor. A scaling factor was calculated from experimental data and used to scale growth rate and branching probability within the computational model based on local ECM density. The factor was calculated by taking the average total vascular length measured for the 2.0, 3.0, and 4.0/ml vascularized constructs and normalizing by the total vascular length for the 3.0 mg/ml construct. Experimental data is presented as the solid points on the graph. This data was then fit to the exponential function described in Eq. 3 (R^2^ = 1.0). The fitted function is presented as the dashed line on the graph.

Qualitative comparison of 3D volumetric confocal image data with the 3D renderings of simulated microvessel growth demonstrated that the computational growth model provided reasonable and realistic descriptions of the experimental data for all matrix densities (Fig. 3 *D–F*). The three-parameter exponential function (Eq. (3)) provided an excellent fit of the experimental data (*R* ^2^ = 0.99) (Fig. 6). The parameters determined by this curve fit were *v_0_* = −0.16, *a_0_* = 5.1605, and *a_1_* = 0.5112. Thus, the inclusion of density-dependent growth and branching resulted in distinct qualitative differences for simulations of angiogenesis across the different matrix density conditions.

The computational model accurately predicted the quantitative morphometric measurements of the vascularized constructs as a function of matrix density. As with the experimental results, simulations predicted a significant reduction in total vascular length as density of the matrix increased (Fig. 4 *A grey*) (ANOVA, P<0.05). Microvasculature predicted by the growth model became increasingly discontinuous as matrix density increased, similar to the trend seen *in vitro* (Fig. 4 *B grey*) (ANOVA, P<0.05). As the density of the matrix increased, the computational simulations predicted reduced branching per unit length (Fig. 5 *A grey*) (ANOVA, P<0.05). Measurements of end points per unit length increased along with matrix density in the simulations (Fig. 5 *B grey*). No statistical difference was detected between any experimental and computational morphometric at each matrix density level by T-test. Additionally, eight out of the eighteen combinations of morphometrics and matrix density passed a TOST-test for statistical equivalence between experimental and computational data: total length at 3.0 and 4.0 mg/ml, branch points at 4.0 mg/ml, end points at 3.0 and 4.0 mg/ml, and normalized end points at 2.0, 3.0, and 4.0 mg/ml (P<0.05).

**Figure 7:**
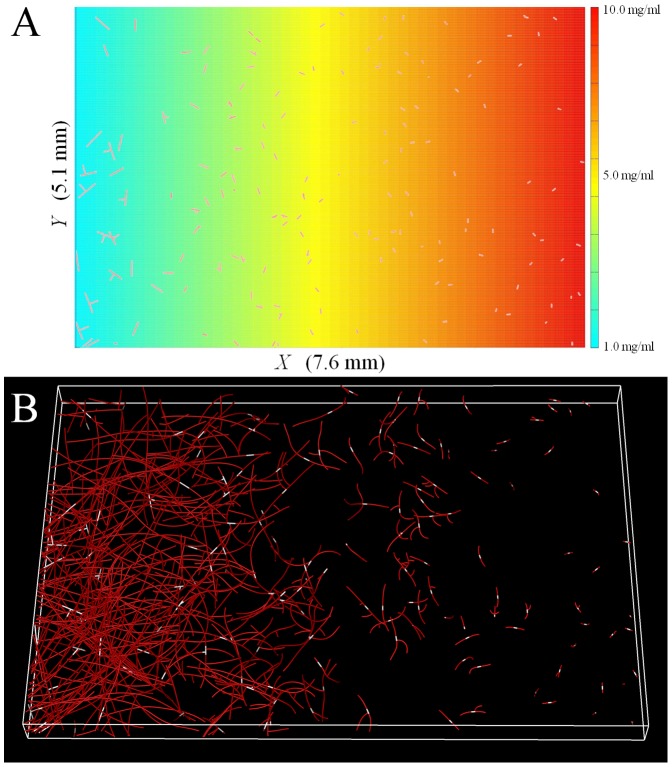
Predictive simulation of angiogenesis within a density gradient. In this simulation, matrix density runs from 1.0/ml to 10.0 mg/ml along the horizontal axis (*x*-axis) while remaining uniform along the other two directions. (A) Z-projection of the matrix density field and the initial microvessel fragments. (B) Growth at Day 6. Initial microvessel fragments are shown in white. The computational framework predicted high amounts of neovascularization in the low density portion of the domain. Growth significantly reduced as density increased along the x-axis. Additionally, vessels that grew towards the low density regions grew at a faster rate than vessels growing towards the high density regions.

In the predictive simulation involving the density gradient, there were high amounts of neovascularization within the low density portions of the gel while growth within the higher density regions was severely reduced (Fig. 7). Neovessels growing down the density gradient (i.e., towards the 1.0 mg/ml region) experienced an increased growth rate and were able to vascularize the gel more effectively. The growth rate of vessels growing up the density gradient (i.e., towards the 10.0 mg/ml region) was severely reduced compared to vessels growing the other direction, and there was very little neovascularization in the high density portion of the gel. In the density plug simulation, vessels were not able to grow into the high density plug, leaving this region of the domain vessel-free while the remainder of the domain became highly vascularized (Fig. S1). The results were similar in the microchannel simulation. The 3.0 mg/ml microchannels were highly vascularized, but very little growth occurred within the regions outside the channels (Fig. S2). Due to the narrow aspect ratio of the microchannels, the resulting microvasculature was aligned along the direction of the channels.

## Discussion

In our experiments, we found that increases in matrix density significantly limited angiogenesis (Fig. 3). Networks cultured in high density matrix had a shorter contour length after the six day culture period (Fig. 4 *A black*), suggesting that increased matrix density decreases neovessel growth rate. Additionally, networks cultured in high density matrix were less divergent than their lower-density counterparts as indicated by a reduction in the number of branch points formed per unit length of growth (Fig. 5 *A black*), suggesting that new sprouts were forming at a slower rate. Finally, an increase in matrix density also resulted in an increase in the number of free ends per unit length (Fig. 5 *B black*). Thus, as the density of the matrix increased, the observation of increased free ends despite a decreased amount of branching suggests that anastomosis occurred less frequently. This may be due to the fact that a slower growth rate reduces the chance of a neovessel finding a potential vessel for anastomosis, resulting in poor network formation (Fig. 4 *B black*).

Our computational model was capable of accurately predicting microvascular network topology as a function of initial ECM density. As the density of the matrix was increased, the computational model predicted neovasculatures with shorter contour lengths, reduced branching per unit length, more free ends per unit length, and reduced connectivity (Fig. 4, 5). For all cases, no statistical difference could be detected between morphometric data from the experiments and computational simulations at a given level of matrix density by T-test. Almost half of the morphometric-matrix density combinations tested positive for statistical equivalence, and many of the P-values were under 10% even if equivalence was not found. Additionally, in almost all cases, the computational mean was within one standard deviation of the experimental mean. These results demonstrate that the computational model was capable of making similar predictions when compared to the experiments across matrix density for all morphometrics, even if statistical equivalence was not found in each case. It should be noted that although our sample number produced high statistical power for the ANOVA tests, PostHoc analysis revealed that the power of the T-tests at this sample number was low (less than 80%) and the number of experiments required to reach a power of 80% is very high in most cases and outside the practical limitations of the study. However, despite these limitations in the statistical analysis, the level of similarity between experimental and computational data presented here satisfies the objectives of the study.

It is instructive to examine the results of this study using an organ culture model of angiogenesis in the context of other studies that have examined the effect of changes in matrix density on morphogenesis of endothelial cell culture models. These cell culture models included HUVECs (human umbilical vein endothelial cells) within a type-I collagen matrix [18], HUVEC-coated microbeads within a fibrin matrix [15]–[17], BPMECs (bovine pulmonary microvascular endothelial cells) cultured on a collagen substrate [19], and BAECs (bovine aortic endothelial cells) cultured on compliant polyacrylamide substrates functionalized with type-I collagen [20]. In all of these studies, increasing the density of the matrix significantly reduced outgrowth and network formation. The organ culture model used in our study differs from the experimental models in previous studies. Microvessel fragments consisting of endothelial cells, pericytes, and basement membrane where cultured in a collagen matrix as opposed to isolated endothelial cells or mixtures or endothelial cells and e.g. pericytes. An advantage of our *in vitro* organ culture model is that it includes multicellular signaling and organization and neovessels with stromal cells. However, a weakness of this system is that it is more complicated which makes it more difficult to isolate contributing factors compared to single cell models. We found similar results to studies with cell culture models in that increasing matrix density reduced angiogenic outgrowth and neovascularization. Vessel fragments are able to form continuous vascular networks resembling *in vivo* microvasculature during culture. This allowed us to analyze the relationship between matrix density and morphometric features of vascular networks during angiogenesis that not typically analyzed in simpler cell culture models, such as branch points and free ends per unit length of growth and network interconnectivity. We also designed and implemented a computational model capable at simulating the organ culture experiments, and we applied this modeling framework to predict other experiments. For example, the method of encapsulating vessel fragments within a collagen matrix would not allow us to culture angiogenic microvessels within a pre-fabricated gradient of matrix density, but we can simulate that experiment (Fig. 7). This simulation predicted a large amount of neovascularization in the low density portions of the domain, and vascularization decreased as density increased along the direction of the gradient.

Our computational results obtained by scaling the rate of growth and branch formation suggests that matrix density alone can regulate the topology of the vascular network by controlling these two aspects of angiogenesis. As we increased the concentration of collagen monomer in our experiments, we created matrices with increased fibril density, more inter-fibril cross-links, and greater mechanical stiffness [31], [33]–[36]. This increase in stiffness renders the matrix more difficult for endothelial cells to deform, degrade and remodel. There are numerous studies that suggest that growth during angiogenesis depends on the cells ability to deform, degrade and remodel the ECM. Increasing the matrix resistance to deformation in vitro, either by increasing mechanical stiffness through the structure and composition of the ECM [17], [18], [20], [37] or by imposing boundary conditions to prevent contraction of the gel [19], [29], [33], results in a decrease in angiogenic growth. These results have been observed using both cell and organ culture models of angiogenesis, and suggest that the rate of angiogenic outgrowth is regulated by the stiffness of the matrix. Califano and Reinhart-King used BAECs (bovine aortic endothelial cells) in compliant polyacrylamide substrates functionalized with type-I collagen to demonstrate that stiffness alone regulates angiogenic growth, independent of the density of ligands available for integrin binding [20]. Additionally, Kniazeva et al. demonstrated that increased fibrin density *in vivo* reduced outgrowth despite the presence of both endothelial and stromal cells [38]. VEGF signaling leads to increased stress fibers and focal adhesions within endothelial cells, mediated through the receptor VEGFR2 and activity of Rho and ROCK (Rho-Kinase) [39]–[41]. These results suggest that angiogenic endothelial cells within the sprout tip may receive positive feedback that promotes the angiogenic phenotype and growth when the cells detect a compliant matrix that they can deform and condition. We were able to simulate this behavior in our growth model by scaling the length of new segments creating during the growth step (i.e., the amount of net growth that occurred over the time step) with respect to local matrix density.

Increasing the matrix density also reduced the rate of branch formation during angiogenesis. Ghajar et al. reported similar observations of reduced sprouting upon increasing the density of the matrix using HUVECs cultured on microspheres within a fibrin matrix [15], [16]. A feeder layer of fibroblasts was cultured on top of the matrix to serve as a source of growth factors. Ghajar et al. proposed that increasing matrix density reduced sprouting by decreasing the porosity of matrix and hindering the diffusion of soluble growth factors [16]. This conclusion was supported by the observation that co-culturing fibroblasts within the matrix along with endothelial cells improved sprouting [16]. In the co-culture model, the fibroblasts acted as pseudo-stroma cells and the length over which growth factors had to diffuse over to reach the endothelial cells was drastically reduced. In our experiments, an organ culture model of microvessels within collagen gel exhibited a similar trend of reduced angiogenesis across increases in matrix density. Endothelial cells within this culture model remain intact as microvessel fragments and retain their association with stroma cells (perictyes) and the basement membrane. Therefore, the diffusive length between neovessel sprouts and the stroma was small. Additionally, the growth media in our cultures was supplemented with VEGF in order to ensure a uniform presence of growth factor throughout the gel, although we did not measure diffusivity in our gels as a function of collagen concentration Nevertheless, the inverse relationship between neovessel sprouting and matrix density observed in the present study suggests the existence of an additional mechanism, independent from chemotaxis, which is responsible for the effects of matrix density on angiogenic sprouting.

Endothelial cells within non-sprouting microvessels are subjected to the combined loading of inter-cellular tractions from neighboring endothelial cells and pericytes and extra-cellular forces from the ECM. The results of several studies suggest that the angiogenic sprouting phenotype of endothelial cells is regulated by a balance between intercellular and extracellular forces [12], [42]–[47]. For example, Ingber proposed that if traction applied to a vessel was large enough to cause a shift in this balance, then endothelial cells change to a angiogenic sprouting phenotype and begin the formation of a new sprout [44]. Additionally, a previous computational study predicted that steep gradients in matrix density would lead to increased stress along the neovessel, resulting in sprouting [45]. Cells can mechanically communicate with each other through compliant, deformable substrates [8]–[10]. As angiogenic sprouts grow, they apply traction along protrusions attached to the ECM through integrins at focal adhesions, which causes local deformation and remodeling. The deformation can be detected by other cells if they are within range of the sprout. A strong deformation signal could cause endothelial cells within other vessels to adopt the angiogenic phenotype and form a new sprout, similar to how deformation signals promote growth as discussed earlier. The amount of deformation that sprouts can generate as well as the range over which cell-generated forces travel depends on the stiffness of the ECM. In stiffer matrix, sprouts produce less deformation, forces are not transmitted as far, and as a result, cells within other microvessels experience less extracellular loading and are less prone to adopt the angiogenic phenotype and sprout into a new branch. Although we use a random-based mechanism for branching in our growth model rather than a deterministic approach, we were able to capture this behavior by scaling the probability of branch formation with respect to local matrix density.

The model outlined in this study incorporated a number of simplifying assumptions, and made use of random processes rather than mechanistic and/or deterministic approaches to represent certain aspects of angiogenesis. These limitations are in part due to the fact that the exact mechanisms as to how the mechanical interaction between neovessels and the ECM affect all the different aspects of angiogenesis and neovasculaturization are not well understood. To describe these mechanisms, additional data are needed on both the deformations experienced by cells locally as well as how cells integrate those signals and respond. It is our hope that we can continue to expand the model incrementally, adding complexity as we gain additional experimental insight. For example, matrix density and fibril orientation were assumed to be constant over the growth period, which does not accurately represent changes in the ECM that are known to occur during angiogenesis. Angiogenic neovessels within our organ culture model alter and condense the matrix via traction forces [12], [48]. However, our current framework does not update physical and material properties of the ECM during the growth period in response to deformation caused by cellular traction forces. As a result, the function for calculating the scaling factor in Eq. (3) is based on initial matrix density rather than current matrix density. However, our focus for this research was to obtain a realistic scaling function to properly represent growth and branching rate as density increased. The scaling function achieved this goal, even though it was calibrated using initial collagen concentration yet was applied to all time points during the simulation. The valid predictions of morphometric data in simulations at different matrix density levels indicate that the assumptions used in our approach were sufficient for our level of analysis. In future work, coupling the growth model with the field theories of continuum mechanics will provide a simulation framework for studying how mechanical forces and ECM structure at the microscale influence the topology of the vascular network. Such a framework could be extended to other morphogenic processes and would allow investigators to establish cause-and-effect relationships between cell-generated forces, matrix deformation and remodeling, the subsequent cellular response, and the morphology of the emerging tissue.

In summary, increasing the density of the ECM significantly reduced angiogenesis and network formation within a 3D organ culture model of angiogenesis. The computational framework outlined in this study was capable of predicting this observed experimental behavior by adjusting neovessel growth rate and branching probability according to local ECM density, demonstrating that these changes in neovessel behavior are responsible for the differences in vascular topology we found. In future studies, this computational framework could be extended in the future by coupling vessel growth with matrix deformation, enabling advanced study of the mechanics of angiogenesis and other morphogenic processes.

## Supporting Information

Figure S1 **Predictive Simulation: High-density cylindrical plug.** (A) Z-projection of the matrix density field and the initial microvessel fragments. In this simulation, a 1.5 mm radius plug of 10.0 mg/ml acellular collagen was placed at the center of the domain. Vessels were seeded within the 3.0 mg/ml region outside of the plug. (B) Growth at Day 6. Initial microvessel fragments are shown in white. There were high amounts of neovascularization within the regions surrounding the plug, but vessels that encountered the high-density plug were unable to grow any further. As a result, the plug region remained vessel-free.
(TIF)

Figure S2 **Predictive Simulation: Vascularized microchannels.** (A) Z-projection of the matrix density field and the initial microvessel fragments. (B) Growth at Day 6. Initial microvessel fragments are shown in white. In this simulation, two 1000 µm microchannels of 3.0 mg/ml collagen seeded with vessels were set up along the *x*-axis. During the simulation, the microchannels became highly vascularized but little growth occurred once the vessels left the channel. As a result, vasculature became aligned along channels as vessels growing along the channel grew at an increased rate.
(TIF)
