# Supplementary figures and images for: Extracellular Matrix Density Regulates the Rate of Neovessel Growth and Branching in Sprouting Angiogenesis
Source: PLoS One. 2014 Jan 22;9(1):e85178. doi: 10.1371/journal.pone.0085178 (PMC3898992; doi:10.1371/journal.pone.0085178)

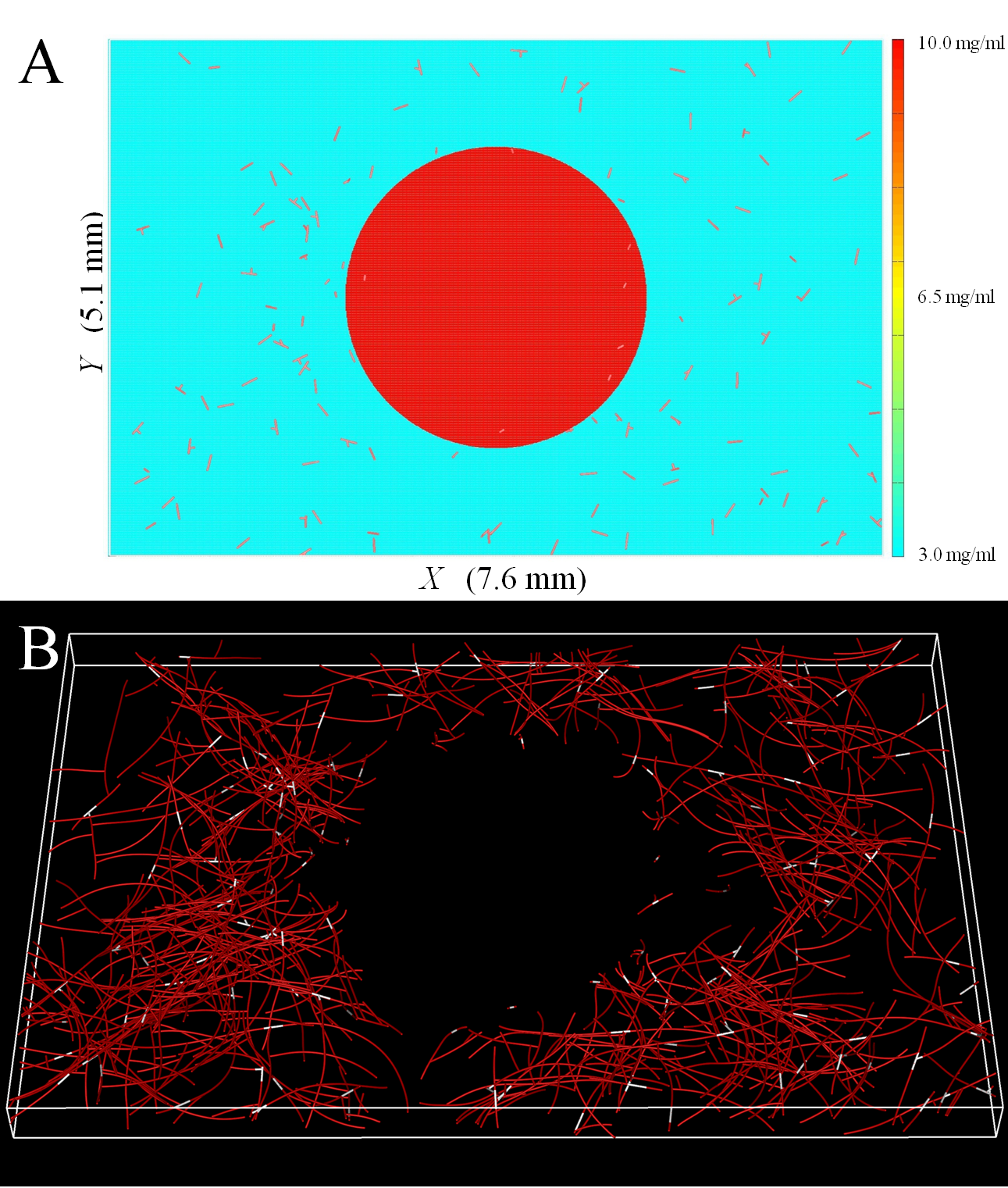

Supplement: Figure S1 — Predictive Simulation: High-density cylindrical plug. (A) Z-projection of the matrix density field and the initial microvessel fragments. In this simulation, a 1.5 mm radius plug of 10.0 mg/ml acellular collagen was placed at the center of the domain. Vessels were seeded within the 3.0 mg/ml region outside of the plug. (B) Growth at Day 6. Initial microvessel fragments are shown in white. There were high amounts of neovascularization within the regions surrounding the plug, but vessels that encountered the high-density plug were unable to grow any further. As a result, the plug region remained vessel-free. (TIF) [file pone.0085178.s001.tif]

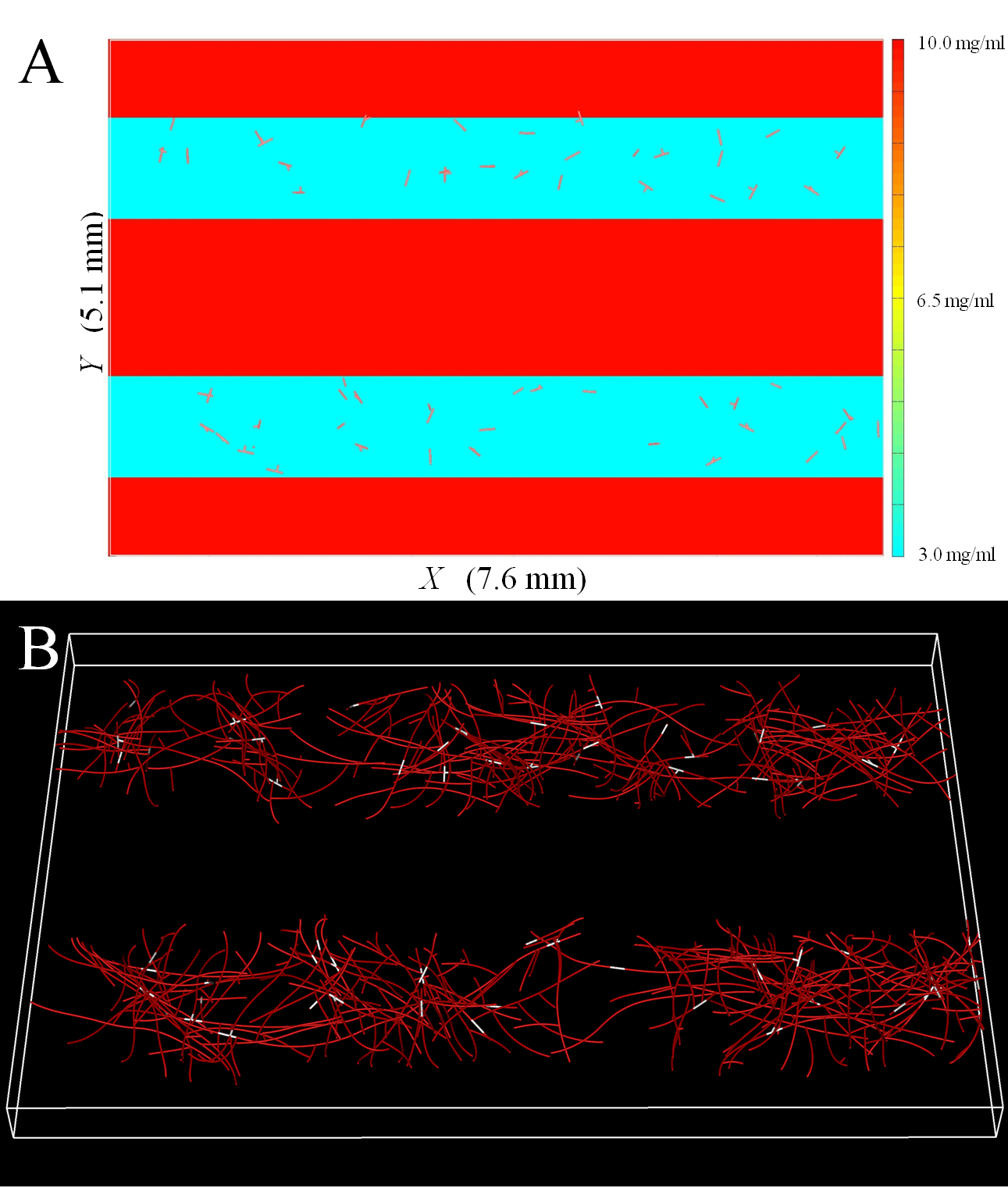

Supplement: Figure S2 — Predictive Simulation: Vascularized microchannels. (A) Z-projection of the matrix density field and the initial microvessel fragments. (B) Growth at Day 6. Initial microvessel fragments are shown in white. In this simulation, two 1000 µm microchannels of 3.0 mg/ml collagen seeded with vessels were set up along the x-axis. During the simulation, the microchannels became highly vascularized but little growth occurred once the vessels left the channel. As a result, vasculature became aligned along channels as vessels growing along the channel grew at an increased rate. (TIF) [file pone.0085178.s002.tif]
